# p53, sex, and aging: lessons from the fruit fly

**DOI:** 10.18632/aging.100101

**Published:** 2009-11-13

**Authors:** Jae H. Hur, David W. Walker

**Affiliations:** ^1^ Department of Physiological Science, University of California, Los Angeles, CA 90095, USA; ^2^ Molecular Biology Institute, University of California, Los Angeles, CA 90095, USA

**Keywords:** Drosophila melanogaster, sexual antagonistic pleoitropy, longevity

The p53 tumor suppressor gene is activated by numerous cellular stressors, including hypoxia and DNA damage and may induce cell cycle arrest or apoptosis depending on the extent of the damage [1].  This capacity has earned p53 the title of ‘guardian of the genome' [2] and in 1993, p53 was voted ‘Molecule of the Year' by *Science* magazine [3].  Since then, the extensive study of p53 at both structural and functional levels has provided greater insight into its role in cancer biology [4].  The fact that p53 functions to suppress cancer means that it is essential.  However, recent studies in a broad spectrum of model organisms, including mice, have shown that not all functions of p53 are beneficial to a long healthspan [5].

## p53

One of the first indications that p53 may have a ‘dark side' was the observation that even a slight constitutive hyper-activation of p53 results in premature aging phenotypes in rodents [6,7].  While adult mice that express a hypermorphic truncated p53 allele show a dramatic reduction in incidence of cancer, they nevertheless have significantly shortened life spans and show many hallmarks of early aging, including loss of body mass and reduced stress resistance [7].  A severe reduction in p53 activity, on the other hand, causes rampant tumorigenesis.   Therefore, p53 may provide an example of antagonistic pleiotropy, where expression in early life is beneficial in preventing cancers, but higher levels of expression can result in, among other effects, detrimental reductions in stem cell pools that maintain organ homeostasis [8].

Recently, studies in *Drosophila melanogaster*, have greatly improved our understanding of the role of p53 in modulating the aging process.   Using tissue-specific drivers and dominant-negative {DN) p53 alleles, a reduction in p53 function in adult neurons was shown to have a beneficial effect on longevity [9].  Interestingly, life spans of flies that have reduced p53 function in adult neuronal cells cannot be further extended by dietary restriction (DR) [9], indicating that a decrease in p53 activity may be a part of the DR lifespan-extending pathway in flies.  Moreover, dSir2, a gene previously implicated in mediating response to dietary restriction [10], interacts directly with and deacetylates p53, inhibiting its function as a transcriptional activator [11].  One consequence of the lowered p53 activity in adult neuronal cells is a reduction in the secretion of *Drosophila* insulin-like peptide 2 (dILP2) without a similar reduction in the secretion of other dILPs [12].  Thus, in addition to its vital role in safeguarding the genome, p53 may also tie together the responses generated by DR and the insulin/IGF-1 signaling pathway.

## Sex

New insights into the roles that p53 plays in animal aging are provided by Waskar et al. in this issue of *Aging*.  Following up on previous studies that have focused on drawing conclusions about general functions of p53 in aging and senescence, Waskar et al. present an examination of the specific roles p53 plays in different sexes and at different developmental stages.  Provocatively, their results suggest that in addition to the developmental antagonistic pleiotropy characteristic of the role of p53 as a pro-apoptotic gene [summarized in 14 and 15], p53 may also function in a sexually antagonistic manner, limiting the life span of adult female flies while promoting the longevity of adult males.

Evidence from rodent studies suggests that increasing p53 function using a hypermorphic p53 allele speeds up aging in mature animals by depleting the pool of stem cells that are necessary to maintain essential functions [6].  Furthermore, manipulations of p53 in *Drosophila* life span studies have primarily been concerned with the effects of reducing p53 function using DN p53 alleles [9,11,12].  Reports of wild type p53 over-expression have been limited to adults and only in neuronal tissue, where it was shown to have little effect on longevity [9].  What effect a tissue-general over-expression of wild type p53 might have in a largely post-mitotic organism has remained an open question.

Weskar et al. make a significant contribution by taking advantage of the *Drosophila* Gene-Switch system that puts transcriptional control of a transgene under temporal control [16,17].  By inducing the Dmp53 transgene in a tissue general manner in adults, the authors show that in female flies, high levels of p53 function results in a reduction of life span.  Accordingly, the expression of a DN p53 in adult females extends life span, and hypomorphic and amorphic mutations of the endogenous p53 result in life span extension and moderate stress resistance. Surprisingly, in some cases, the authors find the opposite to be true in males, with increased p53 levels in adults resulting in longer life spans.

These new findings from Weskar et al. are fascinating, providing ‘food for thought' at several levels. For one thing, their findings hint that male and female longevity may be limited by different physiological processes. The elucidation of the mechanisms that underlie the sexual antagonistic pleiotropy demonstrated in this study will be of great interest both to studies of life span determination and evolution of antagonistic functions within a single gene.

## Aging

Moderation has been a hallmark of the longevity field, from hormetic effects of toxins [reviewed in 19], to dietary restriction [reviewed in 20], to mitochondrial gene manipulations [18,21].  Weskar et al. show that the positive effects of p53 on life span are no different.  In addition to temporal control, the Gene-Switch system allows for control of the magnitude of transgene expression.  Previous reports of life extension using p53 manipulations in flies have focused on adults where the pro-apoptotic effects of p53 over-expression do not interfere with normal development [9]. In the current study, the authors find that even during development, moderate over-expression of p53 can extend life span.  Moreover, unlike adults that show sexual antagonistic pleiotropy, interventions that extend life span in juveniles appear to be beneficial for both sexes.

The slight elevation in p53 may help maintain genomic fidelity during early life, resulting in healthier, longer-lived adults, at the cost of developmental speed and elevated energy requirements.  In addition, increased p53 activity during development may have resulted in a decrease in body mass/size of the animals.   Although the relationship between body size and longevity is complicated [22], this may be worthy of follow-up studies. In addition, an important question that has emerged from this study is whether the beneficial effects of moderate p53 over-expression during developmental stages is the result of a mechanism that is distinct from the genome protective effects of higher p53 levels.

It has also not escaped our attention that p53 regulates mitochondrial electron transport chain (ETC) activity [23].  Our own interest in the relationship between ETC activity and longevity leads us to speculate that alterations in energy metabolism may be important in p53-mediated longevity.  Interestingly, we have observed stronger effects on female life span in our ETC studies [21], though, it is not clear how (or if) this relates to the sexual antagonistic pleiotropy seen by Waskar et al. with p53.

A final thought worthy of consideration is that it is at least possible that the effects observed with neuronal expression of DN p53 transgenes could be due to the transgenic p53 having effects distinct from inhibition of endogenous p53.  The DN p53 has either a mutation in the DNA binding domain, or it is a truncated version of the protein, but these can presumably still carry out some of the functions of p53, such as localization to the mitochondria, or direct regulation of autophagy.  In addition, while the adult fly is usually referred to as a ‘post-mitotic' system, there are dividing stem cells and progeny cells in the gonad, gut, and malphigian tubule [24-28].  Changes in p53 activity could affect the maintenance of these cells and consequently, the life span of the entire animal.
